# Feline Injection-Site Sarcoma and Other Adverse Reactions to Vaccination in Cats

**DOI:** 10.3390/v15081708

**Published:** 2023-08-08

**Authors:** Katrin Hartmann, Herman Egberink, Karin Möstl, Diane D. Addie, Sándor Belák, Corine Boucraut-Baralon, Tadeusz Frymus, Albert Lloret, Regina Hofmann-Lehmann, Fulvio Marsilio, Maria Grazia Pennisi, Séverine Tasker, Etienne Thiry, Uwe Truyen, Margaret J. Hosie

**Affiliations:** 1 Small Animal Clinic, Centre for Clinical Veterinary Medicine, LMU Munich, 80539 Munich, Germany; 2 Department of Biomolecular Health Sciences, Faculty of Veterinary Medicine, University of Utrecht, 3584 CL Utrecht, The Netherlands; h.f.egberink@uu.nl; 3 Institute of Virology, Department for Pathobiology, University of Veterinary Medicine, 1210 Vienna, Austria; karinmoestl@gmail.com; 4 Maison Zabal, 64470 Etchebar, France; draddie@catvirus.com; 5 Department of Biomedical Sciences and Veterinary Public Health (BVF), Swedish University of Agricultural Sciences (SLU), P.O. Box 7036, 750 07 Uppsala, Sweden; sandor.belak@slu.se; 6 Scanelis Veterinary Test Laboratory, 31770 Colomiers, France; corine.boucraut@scanelis.com; 7 Department of Small Animal Diseases with Clinic, Institute of Veterinary Medicine, Warsaw University of Life Sciences—SGGW, 02-787 Warsaw, Poland; tadeusz_frymus@sggw.edu.pl; 8 Fundació Hospital Clínic Veterinari, Universitat Autònoma de Barcelona, Bellaterra, 08193 Barcelona, Spain; albert.lloret@uab.es; 9 Clinical Laboratory, Department of Clinical Diagnostics and Services, Vetsuisse Faculty, University of Zuich, 8057 Zurich, Switzerland; rhofmann@vetclinics.uzh.ch; 10 Faculty of Veterinary Medicine, Università degli Studi di Teramo, 64100 Teramo, Italy; fmarsilio@unite.it; 11 Dipartimento di Scienze Veterinarie, Università di Messina, 98168 Messina, Italy; mariagrazia.pennisi@unime.it; 12 Bristol Veterinary School, University of Bristol, Bristol BS40 5DU, UK; s.tasker@bristol.ac.uk; 13 Linnaeus Veterinary Ltd., Shirley, Solihull B90 4BN, UK; 14 Veterinary Virology and Animal Viral Diseases, Department of Infectious and Parasitic Diseases, FARAH Research Centre, Faculty of Veterinary Medicine, Liège University, B-4000 Liège, Belgium; etienne.thiry@ulg.ac.be; 15 Institute of Animal Hygiene and Veterinary Public Health, University of Leipzig, 04103 Leipzig, Germany; truyen@vetmed.uni-leipzig.de; 16 MRC-University of Glasgow Centre for Virus Research, Glasgow G61 1QH, UK; margaret.hosie@glasgow.ac.uk

**Keywords:** VAAE, FISS, side effect, adverse event, vaccine, immunization, hypersensitivity reaction, anaphylaxis

## Abstract

Vaccine-associated adverse events (VAAEs), including feline injection-site sarcomas (FISSs), occur only rarely but can be severe. Understanding potential VAAEs is an important part of informed owner consent for vaccination. In this review, the European Advisory Board on Cat Diseases (ABCD), a scientifically independent board of feline medicine experts, presents the current knowledge on VAAEs in cats, summarizing the literature and filling the gaps where scientific studies are missing with expert opinion to assist veterinarians in adopting the best vaccination practice. VAAEs are caused by an aberrant innate or adaptive immune reaction, excessive local reactions at the inoculation site, an error in administration, or failure in the manufacturing process. FISS, the most severe VAAE, can develop after vaccinations or injection of other substances. Although the most widely accepted hypothesis is that chronic inflammation triggers malignant transformation, the pathogenesis of FISS is not yet fully understood. No injectable vaccine is risk-free, and therefore, vaccination should be performed as often as necessary, but as infrequently as possible. Vaccines should be brought to room temperature prior to administration and injected at sites in which FISS surgery would likely be curative; the interscapular region should be avoided. Post-vaccinal monitoring is essential.

## 1. Introduction

Vaccination is undoubtedly one of the most effective measures for the prevention of infectious diseases. However, as with other biologicals, vaccine-associated adverse events (VAAEs), including feline injection-site sarcomas (FISSs), can occur [1,2]. The European Advisory Board on Cat Diseases (ABCD), a scientifically independent board of experts on feline medicine from eleven European countries, created this review based on its guidelines on “adverse reactions to vaccination” [1] and “feline injection-site sarcoma” [3] to help veterinarians in the decision making on optimizing vaccination in individual cats.

Vaccination of cats has always received scientific and public attention following the supposition that a range of rare VAAEs can arise [4]. Although VAAEs are believed to be rare, understanding their potential occurrence is an important part of informed consent for owners when deciding about vaccination [5,6]. The greatest difficulty is to obtain data on those VAAEs that manifest months or years after vaccination. In contrast, those VAAEs that occur soon after vaccination (e.g., within days) and/or at injection sites are more easily recognized. Despite VAAEs are being likely underreported in all animal species, VAAEs were more often reported in dogs and cats than in other animals [7].

VAAEs are caused either by an aberrant innate or adaptive immune reaction, excessive local reactions to the vaccine at the inoculation site or by an error in administration (i.e., not in accordance with the vaccine’s summary of product characteristics). Failure in the manufacturing process could also lead to an increased risk of VAAEs, but vigorous quality control for vaccine manufacture makes this extremely unlikely.

A range of clinical signs can follow the application of vaccines, and, in some cases, these might be associated with the vaccination process. However, a causal relationship is often difficult to prove because of inconsistency in the time of appearance of the clinical signs after vaccination as well as the variable clinical appearance of more chronic systemic VAAEs [4]. In terms of quantifying reactions, VAAEs are generally underreported to vaccine manufacturers or national regulatory authorities (where such reporting schemes exist) [8], as many reactions might not be reported by the veterinarian or the owner of the animal. However, in human medicine, substantial case capture, at least for clinically severe VAAEs, has been demonstrated [9]. Underreporting makes it difficult to obtain reliable data on the nature and true incidence of VAAEs in the field.

With one exception, namely the development of feline injection-site sarcomas (FISSs) (subcutaneous sarcomas at an injection site), the number of published studies on VAAEs in cats is limited. In the UK, surveillance work carried out by the Veterinary Medicines Directorate (VMD), including pharmacovigilance reports on VAAEs after drug use in animals, are published regularly [10,11,12,13,14,15,16,17,18], and the pharmacovigilance reporting system in the UK continues to provide an excellent example of practical and effective arrangements for collecting this important information [10]. However, such reporting systems on adverse effects, including VAAEs, do not provide proof of a causal relationship between drug application (e.g., vaccination) and adverse events (e.g., VAAEs), although the association of vaccination with the development of disease is often observed if a close temporal relationship exists with the administration of the vaccine(s) [4,19]. Epidemiological data on the occurrence of disease related to vaccination are used to support the cause–effect relationship. In one substantial survey in the USA, many cases of VAAEs were recorded in cats that were presented to the Banfield Pet Hospitals between 2002 and 2005 [20]. During this period, more than 1.25 million doses of various vaccines were administered to nearly 500,000 cats, and VAAEs within 30 days of vaccination were reported at a rate of 0.52% in the vaccinated cats. The most commonly reported VAAEs were lethargy, anorexia, and fever for three days after vaccination, or local inflammation at the site of injection. In the Banfield Pet Hospital population, the risk of VAAEs was greatest in cats around one year of age and/or increased as the total volume of vaccine and number of vaccines administered concurrently increased [20]. The VAAE rate within three days of vaccination was 0.48% [20], which was greater than the VAAE rate reported for dogs in a similar study [21]. Another study was published in 2002 by the UK Veterinary Products Committee (VPC) [8]. In this study, the number of suspected VAAEs reported to the UK VMD was determined and the percentage of reactions was established based on the company sales data of the total number of vaccines over the same time period. A mean incidence of VAAEs of 0.61 per 10,000 vaccine doses sold for the years 1995–1999 was found. In 2020, 130 reports on VAAEs were received in Switzerland of which 25% (33/130) concerned cats. Many of the reports in cats involved the application of vaccines against feline herpesvirus (FHV), feline calicivirus (FCV), and feline panleukopenia virus (FPV), and some *Chlamydia felis*, in combination with feline leukemia virus (FeLV). Causality assessment between vaccination and the reaction described was considered as being “probable” in 27% and as being “possible” in 44% of all reported cases (all species), demonstrating that the confirmation of a causal relationship is difficult [7]; moreover, demonstration of true causality would need prospective studies with a very high number of participants. Although observational studies have their limitations, such as likely underreporting of the true number of VAAEs and inclusion of only particular types and brands of vaccines, they support the conclusion that VAAEs are rare in veterinary practice [7].

## 2. Non-Specific Systemic Reactions

The non-specific innate immune response can induce mild systemic signs, such as fever and lethargy, and these are the most common VAAEs observed after vaccination [20]. One prospective study did investigate the incidence of VAAEs in cats following vaccination against FPV, FHV, and FCV. Only 9.8% (11/112) of the cats developed a mildly reduced general condition for a few days that was, however, positively correlated with an antibody response to FPV vaccination; no severe VAAEs were noted [22]. These signs are indicative of the vaccine stimulating the immune system [21,22], but, nevertheless, it is preferable that they should minimal impact on the health of the animal. A significant association between VAAEs, including non-specific systemic reactions (e.g., lethargy with or without fever), and the number of concurrently administered vaccines or the total vaccine volume administered was found. Unfortunately, the number of agents vaccinated for and the number of separate vaccine injections per visit were not stated in that paper [20]. In an older study from 1993, significantly more reactions (e.g., lethargy and inappetence) were detected in cats vaccinated with a multivalent vaccine (at that time a new vaccine against FPV, FHV, FCV, and “*Chlamydia psittaci*”) when this vaccine was used concurrently with an FeLV and rabies vaccine, than without FeLV and rabies [23].

## 3. Hypersensitivity Reactions

Vaccination can lead to different types of hypersensitivity reactions, although these VAAEs are rare in cats [20].

### 3.1. Type I Hypersensitivity Reactions

Type I hypersensitivity reactions are the most common and occur when allergens cross-link immunoglobulin E (IgE) molecules that are bound to mast cells and basophils. Such cross-linking triggers the degranulation of these cells and the immediate release of histamines and heparin, followed by generation and release of prostaglandins and leukotrienes [24,25]. This results in increased vascular permeability, tissue edema, cutaneous pruritus, and bronchial smooth muscle contraction. Clinical signs in cats associated with a type I hypersensitivity reaction can include subcutaneous edema (often facial), pruritus, vomiting, diarrhea (watery and/or hemorrhagic), hypersalivation, respiratory distress, and anaphylactic shock [4,26,27]. Data from the UK VPC indicate that anaphylaxis occurs in one of 555,000 vaccinated cats [19]. Such reactions would normally be expected to occur within minutes (20–30 min) of vaccination [28,29]. Vaccines can contain several potential allergens, including adjuvants, preservatives, antibiotics, culture medium proteins, and additives. Usually, it is not clear which vaccine component is responsible for the induction of the IgE-mediated reaction, though inactivated and adjuvanted vaccines are more likely to be associated with this type of hypersensitivity [30]. In dogs, bovine serum albumin (BSA), which is included as a vaccine excipient, is responsible for many of these acute reactions [31,32], and thus, manufacturers have worked to reduce the content of BSA in canine vaccines; BSA might also cause acute reactions in cats. Indeed, in Japan, severe VAAEs after vaccination in cats were examined from 316 cases reported to the authorities during a 15-year period, with 130 cats (41%) showing anaphylaxis, and in 99/130 cats (76%) anaphylaxis resulted in death; it was suggested that high levels of BSA in the commercially available feline vaccines indicated insufficient purification [33].

Such reactions might occur on the occasion of the first vaccination of a kitten; however, an animal that developed a hypersensitivity reaction to a vaccine does not necessarily develop a reaction to the next vaccine [28]. It is prudent to inform the owner about the risks of subsequent vaccines and to take some measures to reduce the risk of further hypersensitivity. Firstly, the need for each additional vaccination must be weighed against the risk of infection. Also, the number of vaccine antigens administered at one visit should be reduced whenever possible, as a greater risk of developing VAAEs has been associated with a higher number of applied vaccines [20]. If possible, a different vaccine formulation (or product from a different manufacturer) is recommended instead of the one associated with the VAAE. If appropriate, non-adjuvanted modified live virus (MLV) vaccines should be used instead of adjuvanted inactivated (killed) products because the latter are more likely to be associated with hypersensitivity reactions [30]. Subcutaneous and not intramuscular inoculation of the vaccines will reduce the risk of direct uptake of vaccine components into the systemic circulation. Premedication with antihistamines can be administered at least 15 (up to 30) minutes prior to vaccination. After vaccination, predisposed animals should be kept under observation in the clinic for a few hours and watched then at home by the owners [4,27,34].

### 3.2. Type II Hypersensitivity Reactions

Type II hypersensitivity reactions are considered an autoimmune reaction and the result of binding of antibodies to host cells. Cell-bound antibodies can fix and activate the complement pathway leading to lysis of cells. Different effector cells can bind to these cell-bound antibodies via Fc receptors leading to cell-mediated damage of the host cells [30]. In dogs, vaccination has been linked to some autoimmune disorders, including immune-mediated hemolytic anemia (IMHA) and thrombocytopenia (IMTP), polyneuritis, and polyarthritis [19,35]. However, there are no reports suggesting such an association in cats.

Cats inoculated with parenteral FPV, FHV, and FCV vaccines were shown to develop antibodies against cellular antigens of Crandell Rees Feline Kidney (CRFK) cells [36,37]. Since vaccine strains are commonly produced in CRFK cells, cellular antigens might be included in the vaccine. Antibodies recognizing CRFK cells were shown to also react with feline renal extracts. However, neither clinical signs nor abnormalities in urinalysis, or biochemical blood parameters were observed in any cat. In one study, frequent or annual vaccination was identified as one of two risk factors for the development of chronic renal disease (CRD) in geriatric cats [38]. In conclusion, these studies suggest a potential etiological role for these antibodies, induced after primary and booster vaccinations, in causing interstitial nephritis, but definite causal proof is lacking.

### 3.3. Type III Hypersensitivity Reactions

Type III hypersensitivity reactions are rarely documented in cats in association with vaccination [20]. Type III reactions are characterized by antigen–antibody complexes that induce an acute inflammatory response after deposition in the capillary beds of certain tissues [24]. Polyarthritis after FCV infection, which is characterized by lameness and fever, that also occurs sometimes after vaccination has been suggested to represent a type III reaction. However, co-infections with FCV field virus or, rarely, vaccine virus seem to be the main causes of this syndrome [39,40].

### 3.4. Type IV Hypersensitivity Reactions

Type IV hypersensitivity reactions (delayed-type hypersensitivity) are also rarely documented in cats in association with vaccination [20]. They are considered to result primarily from cell-mediated, cytotoxic immune responses, rather than from an antibody response to a specific antigen. Thus, type IV hypersensitivity reactions differ fundamentally from type I, type II, and type III hypersensitivity reactions [19,41]. Upon exposition to a sensitizing antigen, memory Th1 cells are produced; re-exposure leads to the reactivation of these cells and an inflammatory response (typically after 24 to 72 h) by the release of proinflammatory cytokines [41]. Granuloma formation might occur [24], characterized by accumulations of immune cells, which is the hallmark of type IV hypersensitivity [42]. Granulomatous inflammation after vaccination has also been demonstrated in cats [41].

## 4. Immunosuppression

Studies on vaccine-induced immunosuppression have been performed mainly in dogs. Depression of cellular and innate immunity was demonstrated in dogs two weeks post-vaccination [43]. However, this was not a general observation in other studies. It has been suggested that vaccine-induced immunosuppression could also occur in cats, but this is likely to be rare [19]. An outbreak of salmonellosis in cats was observed following the use of a high-titer modified-live FPV vaccine; although not proven, mild immunosuppression induced by vaccination could have facilitated the development of fatal salmonellosis in kittens carrying the pathogen [44].

## 5. Mild Local Reactions at the Injection Site

Mild cutaneous reactions at the injection site are not uncommon in cats [20]. These include swelling, irritation, erythema, loss of hair, pain, and rarely abscess formation. These reactions are more often seen with inactivated and adjuvanted vaccines [19]. An inflammatory reaction at the site of vaccination is in fact part of a desired innate immune response, especially in inactivated vaccines. It is induced by the release of cytokines and chemokines by immune cells and is required for the subsequent development of an appropriate adaptive immune response. However, as cats are prone to develop FISSs, all lumps should be closely monitored [3,45].

Use of a recently introduced non-adjuvanted vaccine against FHV, FCV, FPV, and FeLV formulated in reduced volume (0.5 mL) with the same antigen content as the conventional 1 mL presentation caused fewer local events, while keeping the same immunogenicity as the corresponding 1 mL vaccine, and thus, might help to reduce the incidence of VAAEs [46], although further prospective work is needed to confirm this.

## 6. Feline Injection-Site Sarcoma (FISS)

In cats, the most serious VAAE is the occurrence of invasive sarcomas (mostly fibrosarcomas), so-called FISSs, that can develop within the skin at sites of previous vaccination. A task force was instituted in the USA and is regularly updated to help veterinarians to understand, manage, and prevent these tumors [47,48]. In fact, it was mainly the occurrence of FISSs that increased the concern among cat owners about the safety of vaccines in general and rekindled the discussion on VAAEs in cats.

### 6.1. Epidemiology

Around 1990, an increased incidence of tumors in cats that developed at injection sites was first reported in the USA [49,50]. This observation was connected to an increased use of rabies and FeLV vaccines [50,51]. Consequently, these tumors were first called feline “vaccine-associated sarcomas”. However, the subsequent finding that other non-vaccinal injectables could also be associated with this type of tumor has led to the reclassification of these neoplasms as “feline injection-site sarcomas”. It has been suggested that injections in cats induce repeated local irritation with chronic inflammation, which can act as a precursor to local neoplastic transformation of stromal cells. These tumors seem to be relatively unique to cats [52], although comparable tumors have been reported in ferrets [53] and rarely in dogs [54]. Still, FISSs are extremely similar to soft tissue sarcomas in dogs and humans at the level of gene expression [55].

FISSs occur at sites typically used for vaccination and injections, such as the interscapular region, the lateral thoracic or abdominal wall, the lumbar regions, and in the area of the semimembranosus and semitendinosus muscles in the hindlimbs. FISSs are most commonly located in the subcutis, but also can occur intramuscularly [56,57,58]. FISSs can develop as early as four months and up to two to three years after an injection [59].

In the last 20 years, an epidemiological association has been demonstrated between vaccinations and the later development of FISSs [51,58,60,61,62,63]. The incidence of FISS has been estimated to be one to four in every 10,000 vaccinated cats in the USA [64,65,66], and the ratio of injection-site to non-injection-site sarcomas increased from 0.5 in 1989 to 4.3 in 1994 [67]. In one study from 1998 to 2000 in the USA and Canada, reported rates were 0.3 cases of FISS per 10,000 vaccines and 11.8 post-vaccination inflammatory reactions per 10,000 vaccinations in cats [65]. If inflammatory reactions are a necessary prelude to FISSs, then these rates suggest that one in 35 to 40 inflammatory reactions develop into FISS. During 2008–2013, in Poland, the prevalence of FISS was estimated to be 16 in 10,000 cats in general practice and 85 in 10,000 cats in practices specialized in oncology [68]. In the UK, the incidence of FISS seems to be relatively low (in 2007, incidence risk of FISS per year was estimated to be one in 16,000–50,000 cats registered by practices, one in 10,000–20,000 cat consultations, and one in 5000–12,500 vaccination visits) [69]. One reason for the low rate might be that rabies vaccination is not a routine procedure for cats in the UK. One study in Canada compared the annual prevalence of feline post-vaccination sarcomas among 11,609 feline skin mass submissions from 1992 to 2010 and revealed no decrease in disease prevalence or increase in the age of affected cats in response to the change in vaccination formulation (introduction of recombinant non-adjuvanted rabies (and later FeLV) vaccines) or recommended changes in feline vaccination protocols (e.g., calling for more selective use of FeLV vaccination and less frequent rabies vaccination) [70]. In contrast, studies in Switzerland demonstrated a marked drop in the relative frequency of fibrosarcoma diagnoses from 2005 to 2014 [57].

### 6.2. Pathogenesis

Despite extensive research on the pathogenesis of FISS, there is no definitive causal relationship that explains their occurrence nor a direct link with vaccination. The most widely accepted hypothesis suggests that chronic inflammatory reaction at the site of injection provides a trigger for subsequent malignant transformation [41]. The mechanism by which the chronic inflammatory reaction might cause tumor formation is not fully understood. Growth factors can promote proliferation, can induce malignant transformation, and can also be involved in the regulation of angiogenesis. Overexpression of growth factors and oncogene activation have been demonstrated in cats with FISS and are suspected to play a role in tumor development [71,72,73]. Fibroblasts can undergo neoplastic transformation through different mechanisms, such as activation of oncogenes and inactivation of tumor suppressor genes. Chronic inflammation can induce production of free radicals and metabolites that cause DNA damage and mutations, acting as an initiator of carcinogenesis. The environment provided by chronic inflammation, coupled with a genetic predisposition, alters the susceptibility to carcinogenic injuries [74]. One study showed that cyclooxygenase-2 (COX-2) is expressed within FISSs and that there is a close relationship between COX-2 expression and the degree of inflammation [75]. Generally, COX-2 participates in the synthesis of arachidonic acid derivatives, including prostaglandin E2, which is related to carcinogenic processes [74,76]. Overexpression of COX-2 is associated with tumor proliferation and invasion, inhibition of apoptosis, suppression of immune surveillance, and angiogenesis [76]. Activation of the nuclear factor-kappa B- (NF-κB-) signaling pathway can target genes associated with tumor progression and up-regulate expression of tumor-promoting cytokines and survival genes in tumors [77]. Additionally, high expression rate of nuclear NF-κB p65 in FISSs and dose-dependent inhibitory effects on the growth of FISSs’ primary cells treated with NF-κB inhibitors suggested a role of NF-κB in FISS development [77]. Activation of the Janus kinase-signal transducer and activator of transcription 3 (STAT3) also might play an important role in the tumorigenesis of FISS [78]. Interestingly, it was shown via immunohistochemistry that the expression of estrogen receptors in FISSs, but not the expression of progesterone receptors, correlated with clinical and histopathological aspects (mitotic index and degree of pleomorphism) and thus, estrogen receptors expression could influence tumor growth [79].

It has been suggested that especially adjuvanted vaccines would be linked to the development of FISS due to the more intense local inflammation associated with such products. However, in a prospective multi-center case–control study from 2003, no specific vaccine or vaccine brand could be incriminated in the formation of FISS [62]. Adjuvants are mostly added to enhance a pro-inflammatory reaction at the site of injection, which is intended and a necessity when applying a killed agent in order to trigger the required immune response. Adjuvants, such as aluminum, have been identified in histological or ultrastructural studies of FISS biopsy samples [61,80,81], and it was thought that these adjuvants originated from adjuvant-containing vaccines. Traces of adjuvants can be seen in the inflammatory reaction, specifically accumulated within macrophages or multinucleate giant cells, as well as in histological sections of FISSs in the transformed fibroblasts [61]. Intracellular crystalline particulate material was found in an ultrastructural study in five of 20 investigated FISSs, and in one of the five cases was identified as aluminum-based material [81]. One study investigated the degree of inflammation after vaccination with adjuvant-containing vaccines versus vaccines without adjuvants. It showed that multi-component vaccines containing the recombinant canarypox-vector FeLV cause less inflammation at the injection site than multi-component vaccines with FeLV and an adjuvant. Three groups of 15 cats were injected with a single dose of one of the three vaccines or saline as a negative control; cats in group A received a non-adjuvanted recombinant canarypox-vectored FeLV vaccine; cats in group B received an inactivated FeLV vaccine with a lipid-based adjuvant; cats in group C were vaccinated with an inactivated FeLV vaccine adjuvanted with an alum-Quil A mixture. On days 7, 21, and 62 post-vaccination, significantly less inflammation was associated with administration of the non-adjuvanted recombinant canarypox-vectored vaccine in group A compared to groups B and C. The inflammation was most severe in cats of group C receiving the aluminum-based adjuvant. Those cats receiving adjuvanted vaccines had evidence of residual adjuvant material accumulated within macrophages even at 62 days post-vaccination [41].

Although the association of adjuvants and more pronounced tissue inflammation has been shown, the association between inflammation and tumor formation is more difficult to demonstrate. It has been suggested that inflammatory cells have an impact on cancer development by increasing the invasive capacity, angiogenesis, and motility of tumor cells [82]. One paper evaluating metallothionein expression in FISSs, demonstrated an association between Ki67 index (a marker of cell proliferation) and tumor grade and inflammatory score in FISSs, suggesting that inflammation plays an important role not only in pathogenesis but also in tumor progression [83].

At first, only rabies and FeLV vaccines were identified as risk factors [51,58,64], but subsequently other vaccines, including vaccines against FPV, FHV, and FCV, also were shown to be associated with the development of FISS in some cases [58,59,64,84,85,86]. In addition to vaccines, injections of long-acting drugs, e.g., glucocorticoids, penicillin, lufenuron [59,62,87,88], cisplatin [89], and meloxicam [90] have been associated with sarcoma formation. In one study, the frequency of administration of long-acting corticosteroid injections (dexamethasone, methylprednisolone, and triamcinolone) was significantly higher in cats with FISS in the interscapular region than in control cats [59]. Fibrosarcomas were also reported at the site of a deep, non-absorbable suture in one cat [91], at the site of a retained surgical sponge in the abdomen of one cat [92] adjacent to the site of microchip implantation in two cases [93,94], and associated with a subcutaneous fluid port device in one cat [95]. This suggests that any inflammatory reaction independent of the stimulus, theoretically, could have the potential to lead to the development of FISS through triggering uncontrolled proliferation of fibroblasts and myofibroblasts, which, in some cases, results in malignant transformation.

Many causes of inflammation could be associated with FISS development, and FISSs can occur in animals following vaccination with inactivated, recombinant, modified live vaccines as well as with non-vaccine injections, e.g., in the UK, the VMD received notifications of FISSs in cats associated with different vaccines. However, these reports did not take the number of injected vaccines into account [10,11,12,13,14,15,16,17,18]. In a prospective case-control study conducted at the University of California, Davis, USA, it was demonstrated that, among injections, vaccines were more frequently associated with FISS development compared to other compounds; amongst vaccines, the risk was significantly higher when adjuvanted vaccines were used [59]. One study in Switzerland showed a marked decrease in the number of FISSs from 2005 to 2014, when compared to older studies in the same region [96,97], and the authors of the paper concluded that the introduction of a non-adjuvanted FeLV vaccine in 2007 might have accounted for the decrease [57]. However, the decrease in FISS incidence was in no relation to the non-adjuvanted vaccines sold at the time, and the timespan that is necessary for FISS development was not taken into account. Other factors were not addressed in the study, such as increased awareness of good vaccination practice, not injecting cold vaccines, and vaccinating only if necessary (e.g., Switzerland became rabies-free in 1999).

One study compared associations between vaccine types and other injectable drugs with the development of FISS in a case–control study of 181 cats with soft tissue sarcomas (cases), 96 cats with tumors at non-vaccine regions (control group I), and 159 cats with basal cell tumors (control group II). There was an association between the administration of various types of vaccines and other injectable products (e.g., long-acting corticosteroids) and FISS development. Of 192 sarcomas, 101 had received vaccinations at the site of tumor development during the preceding three years, and 23 had received other injections at that site [59]. This study also showed that adjuvanted-inactivated rabies vaccines were significantly more commonly associated with FISS development than recombinant rabies vaccines in the broad rear limb region; of 35 vaccinated cats with sarcoma on the hindlimb, 25 had received inactivated (predominantly rabies) vaccines, seven cats had received MLV vaccines (FPV, FHV and FCV), and only one cat had received a recombinant rabies vaccine. However, these findings also indicate that no vaccines were risk-free and that other factors also can be associated with the development of FISS [59]. The latter finding is also supported by the data from the UK VMD receiving notifications of FISSs in cats vaccinated with MLV, inactivated, and recombinant vaccines [10,11,12,13,14,15,16,17,18].

A possible role of FeLV and its mutant feline sarcoma virus (FeSV) in the development of FISS has not been demonstrated by the presence of either FeLV or FeSV in tumors [98]. One study from Brazil reported positive immunohistochemical staining for FeLV p27 antigen in nine of 21 FISSs [99]; however, the method used (polyclonal antibodies for histology) has most likely low specificity and thus the significance of the results is doubtful. Furthermore, no other viruses, including feline immunodeficiency virus, feline foamy virus, polyomaviruses, or papillomaviruses, were detected in tumor tissues [100,101,102,103]. Additionally, no evidence was found for the replication or the expression of endogenous retroviruses being involved in FISS development [101,103].

The observation that only few cats develop FISSs after vaccination suggests that there might be a genetic predisposition. It has been proposed that there is a higher incidence of FISS in siblings of affected cats, and that some cats tend to develop more than one FISS. Alterations with unknown relevance, such as hyperploidy [104], translocations [105] and triploidy [106] of oncogene and tumor suppressor loci have been found on extra chromosomes and monosomic chromosomes in affected cats. Mutations have been identified in the tumor suppressor gene p53, which is implicated in cancer initiation and progression in sarcoma tissue of cats with FISSs [107,108,109,110,111]. A case-control study (50 domestic shorthair cats with a confirmed diagnosis of FISS and 100 disease-free matched controls), investigating a possible association between polymorphisms in the genomic sequence of the feline p53 gene and a predisposition to FISS, found a strong association between FISS and the presence of specific nucleotides at two of the polymorphic sites [112]. However, another study, conducted in Germany, could not reproduce these findings and observed no association with the polymorphisms described [113], so their significance is unknown.

### 6.3. Clinical Signs

**Figure 1:**
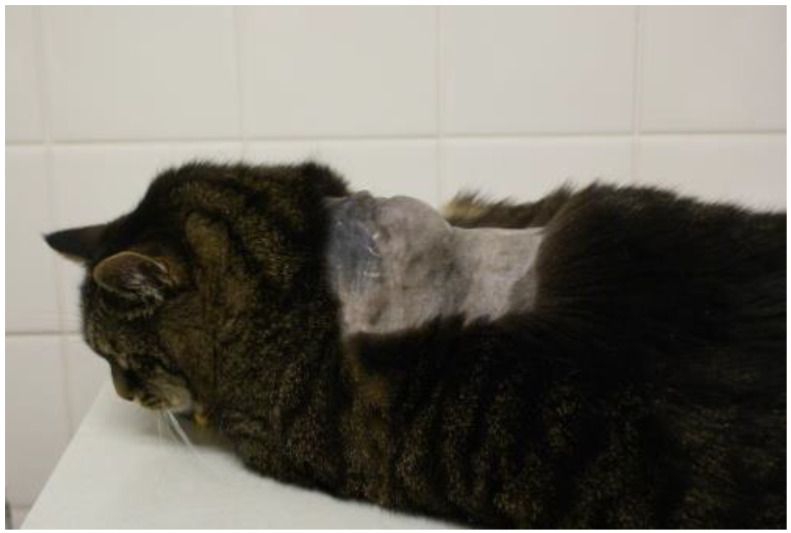
Cat with feline injection-site sarcoma. Courtesy of Johannes Hirschberger, LMU, Munich, Germany.

FISSs are tumors characterized by invasive local growth in the subcutis (rarely within the muscles) (Figure 1), often with a spread along fascial planes [114]. Most FISSs are fibrosarcomas [67], but other malignancies, such as osteosarcomas [115], chondrosarcomas [58], rhabdomyosarcomas [58], malignant fibrous histiocytomas [58,115], and myofibroblastic sarcomas [56] have also been rarely described. FISSs are usually firm, indolent, seemingly well-circumscribed, subcutaneous masses that are often not freely moveable. FISSs behave more aggressively than sarcomas at other sites [58]. The rate of metastasis ranges from 10% to 28% [116,117]. The lung is the most common site of metastasis, followed by regional lymph nodes and abdominal organs, such as kidney, spleen, intestine, and liver [118,119].

**Figure 2:**
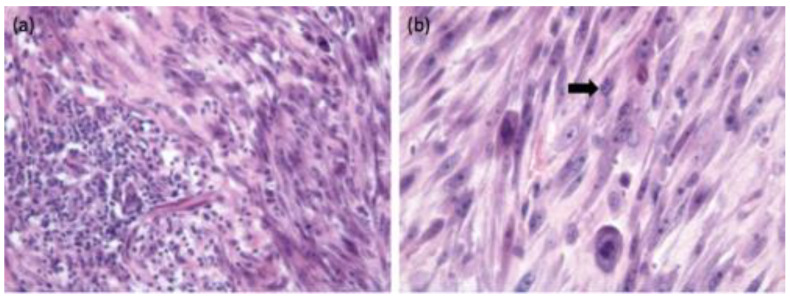
Histological sections of a 2 cm diameter mass removed from the lateral thorax of a 13-year-old domestic shorthair cat. A similar interscapular mass had been removed from this cat two months previously. (**a**) A focus of lymphoplasmacytic inflammation is contained within the surrounding sarcoma. (**b**) Higher magnification of the neoplastic tissue reveals a pleomorphic population of neoplastic spindle cells with occasional giant nuclei and irregular mitotic activity (arrow). Hematoxylin and eosin stain. Pictures provided by Michael Day, Bristol Veterinary School, University of Bristol, United Kingdom.

In computed tomography (CT), common features of FISSs are marked local invasiveness into the musculature and heterogeneity of the tissue in the periphery of the neoplasia [120]. In contrast to fibrosarcomas in other areas, FISSs have different histological characteristics, typically with perivascular infiltration of lymphocytes and macrophages at the tumor periphery, a central area of necrosis, inflammation, and local infiltration of tumor cells (Figure 2) [67,81].

Prognosis of FISS is most importantly influenced by the size of the tumor [121], and radical excision is crucial for a favorable outcome, and thus, prognosis is primarily influenced by the tumor site and its accessibility to surgery and the possibility of obtaining tumor-free margins [45,86,122,123]. Therefore, pre-operative diagnostic imaging (contrast-enhanced CT and/or magnetic resonance imaging (MRI)) is extremely important to determine the true extent of the tumor [124]. Prognosis is better if, in addition to radical surgery, further therapeutic options such as radiotherapy [118,122,125,126,127] or immunotherapy [128,129,130] are applied. In addition, neutrophil-to-lymphocyte ratio, white blood cell count and neutrophil count were determined as prognostic parameters for local recurrence of FISSs [131].

### 6.4. Prevention

Prevention consists of three general considerations. First, injections in cats should always be administered at sites in which surgery (such as amputation of a limb or excision of lateral abdominal skin) would likely lead to a complete cure with the least complicated surgical procedure. Second, general recommendations to reduce the inflammatory reaction at injection sites should be followed, such as avoiding the administration of irritating substances. And third, it is advised to vaccinate as often as necessary, but as infrequently as possible (e.g., according to the principles of current vaccination guidelines, such as triennial vaccination for FHV and FCV in cats with low risk of exposure, avoiding FeLV vaccination in already FeLV-infected cats, or FPV vaccination in cats with pre-existing antibodies against FPV).

In general, injecting distally in the limb aids in the treatment of any subsequent FISS (by limb amputation) because these tumors are very difficult to excise completely and often recur locally after resection [63]. Administration of vaccines (or other injections) between the scapulae is generally contraindicated because tumor resection is almost impossible in this location. To assess the acceptance of the Vaccine-Associated Feline Sarcoma Task Force of the American Association of Feline Practitioners (AAFP) recommendations that include not injecting vaccines interscapularly (published in 1999) by veterinarians, a study including 392 cats with FISSs compared the anatomical locations of tumors between cases with FISSs diagnosed before and after the publication of these recommendations [47]. Comparing the prevalence of cases arising before and after the publication of the vaccination recommendations, the proportions of FISS significantly decreased in the interscapular (53% to 40%) and right and left thoracic (10% to 4% and 9% to 1%, respectively) regions, whereas the proportion of FISS significantly increased in the right thoracic limb (1% to 10%), in the combined regions of the right pelvic limb with the right lateral aspect of the abdomen (13% to 25%) and in the left pelvic limb with the left lateral aspect of the abdomen (11% to 14%). Thus, despite publication of the vaccination recommendations, a high proportion of tumors still developed in the interscapular region. There was also an increase in lateral abdominal FISSs, which could be attributable to an aberrant placement of injections intended for the hindlimbs. Thus, veterinarians are complying with vaccination recommendations to some extent, but only the administration of vaccines as distally as possible on a limb would allow for complete surgical margins if limb amputation is required [132]. Data in Europe show a similar situation. In a study examining the location of FISS in cats presented to the oncology service at the Clinic of Small Animal Medicine of the LMU Munich, Germany, most FISSs still occurred between the scapulae (40%), followed by the right (19%), and left thoracic walls (13%) [133]. A study from Brazil, investigating the anatomical location of biopsy samples compatible with FISSs submitted between 2007 and 2017 found that 35% of the tumors were located on the thoracic wall, 29% in the flank, 21% in the interscapular region, and 15% in the limbs [134]. A cross-sectional study in the UK determined cat owners’ attitudes towards surgical treatments of different anatomical regions: less than half of the owners (39%) would pursue surgery regardless of tumor site; 1% would not pursue surgery; of the remainder, respondents would not allow amputation of the forelimb (20%), hindlimb (15%), or tail (15%). On the other hand, the majority of respondents were willing to travel up to 100 miles for radiotherapy or chemotherapy (66 and 69%, respectively) [135]. Thus, owner education by the veterinarian explaining optimum treatment options is important.

Unfortunately, there is still a lack of information to provide evidence-based vaccine site recommendations. The majority of safety and efficacy data come from licensing studies in which vaccines are administered subcutaneously in the interscapular region, which should not be used for any injection in field cats. Radical surgical resection of FISS including margins of at least 3 cm, but preferably 5 cm [123] is associated with the highest response rate and long-term survival [117]. With this in mind, the AAFP panel on vaccination guidelines conducted an informal survey of veterinarians whose practices focused on radiation (12), surgical (36), and medical (44) oncology for opinions on what the preferred vaccination sites should be [45]. These experts agreed that distal to the stifle followed by distal to the elbow were their preferred sites; nearly as popular was the tail. Respondents frequently commented that vaccines should be administered as low on the leg as possible. They added that vaccination of cats resting in a crouched position often results in inadvertent injection of the skin fold of the flank, resulting in tumors that are difficult to resect [45]. This is reflected in a study which observed an increase in lateral abdominal injection-site sarcomas since the publication of the Vaccine-Associated Feline Sarcoma Task Force vaccination recommendations in 1999 [123]. Based on this, the AAFP recommends distal limb injection to facilitate amputation with 5 cm margins in two fascial planes in FISS cases [6]. Ventral abdominal subcutaneous injections also have been used because of the perceived relative ease of tumor removal without the need for amputation. However, the need to remove two fascial planes and 5 cm margins would still necessitate aggressive tissue removal from the abdomen and abdominal cavity [6]. Tail vaccination has also been reported as being well tolerated and elicited acceptable antibody responses when compared to vaccination in the distal limbs [136]. However, to facilitate 5 cm margins in case of FISS removal, vaccinations must be administered in the distal tail, something that might not be practical for most clinicians [6]. Further studies should be performed to confirm that this would be an alternative option leading to similar vaccine-induced protection rates.

In addition to considering appropriate injection sites, post-vaccination monitoring plays an important role. Vaccination sites should be noted in clinical records [5,6], and veterinarians should instruct their clients to monitor the vaccination (or injection) sites for swelling or lumps so that potential FISSs can be detected early, and thus can be removed successfully. Veterinarians should be aware that **any skin or subcutaneous mass** in a cat requires further diagnostic and interventional approaches. At the very least, owners should be instructed in relation to the “**3-2-1**”-rule: incisional wedge biopsies or total removal and histological examination of any mass is warranted if the mass is still present **three** months after vaccination or if the mass becomes larger than **two** cm in diameter or if the mass is increasing in size **one** month after vaccination. Fine-needle aspirates might not provide diagnostic cellular tissue, whereas excisional biopsies rarely meet the appropriate margins (5 cm in two fascial planes) as required in the case of FISS, thus increasing the morbidity and mortality risks associated with sarcoma invasion [6]. In general, diagnostic investigation is warranted when any cutaneous mass is noted in a cat.

Concerning general recommendations to prevent inflammatory reactions at injection sites, there are a few rules to follow. Generally, cats should receive as few subcutaneous injections as possible. Intramuscular injections in cats should be avoided because intramuscular tumors develop with a similar frequency but are more difficult to detect early. Whenever feasible, cats should receive drugs orally or intravenously. The subcutaneous injection of especially long-acting irritating substances (such as long-acting glucocorticoids) should be avoided. One study examined the potential risk factors when administering vaccines [62], and few factors were associated with the development of FISS. It was observed that the size of the needle and the syringe, the velocity of injection, and whether manual pressure was applied after injection or not, played no role. In contrast, the temperature of the vaccine made a significant difference, with cold vaccines being associated with a higher risk of FISS development than vaccines at room temperature [62]. Thus, vaccines should be taken out of the refrigerator and be brought to room temperature before injection, e.g., by keeping the vial in the palm of your hand for a short time, but should be kept only briefly out of the refrigerator to avoid reduction in vaccinal efficacy. For lyophilized vaccines, the diluent can be kept out of the refrigerator for a longer period. In addition, multi-dose vaccine vials, i.e., ten doses of the same vaccine within one vial, that are only rarely used in cats nowadays, were associated with a higher risk of FISS development [62].

Regarding the risk of FISS development, intranasal vaccines are to be preferred over injectable vaccines in cats if similar efficacy has been demonstrated. However, in most countries, only injectable vaccines are available. Therefore, the vaccines that are preferred are those that cause the least subcutaneous inflammatory reaction.

Considering injectable vaccines, there has been much discussion of whether non-adjuvanted vaccines should be generally preferred over those containing adjuvant, if available and with proven similar efficacy. However, currently there is insufficient information to make definitive recommendations on the vaccine type [137].

The current knowledge is as follows:

- It is a fact that every injectable vaccine can cause FISS [59,62];
- It is a fact that that vaccines containing adjuvant cause more inflammation than vaccines without adjuvants [41];
- It has been reported that inflammation might contribute to FISS development [83];
- There are conflicting data whether vaccine adjuvants truly are associated with increased risk of FISS development [59,62];
- It is currently difficult to truly compare vaccine efficacy studies because of differences in study designs.

Therefore, there is insufficient information to make definitive recommendations on the vaccine type and it is important to consider efficacy data. Where efficacy studies have shown higher protection rates in cats vaccinated with adjuvanted vaccines versus non-adjuvanted vaccines, the former are preferable in cats at high risk of infection. Otherwise, given the current state of knowledge, if vaccines without adjuvants have been proven to be equally effective as adjuvant-containing ones, it is reasonable to suggest that vaccines without adjuvants should be preferred, at least until the pathogenesis of FISS is better understood.

Finally, to prevent the development of FISS, cats should be vaccinated only as frequently as necessary. Therefore, long vaccination intervals should be applied in adult animals where possible. Although all vaccines are licensed with specific vaccination intervals, ABCD guidelines [138] sometimes suggest longer intervals depending on the cats’ lifestyle. No non-core vaccines, such as FeLV, should be administered to cats without an exposure risk. In addition, immune cats should not be vaccinated (e.g., if presence of FPV antibodies is demonstrated as FPV antibodies are correlated with protection) [22,139]. This clearly demonstrates the necessity of individual vaccination schedules [2,6].

## 7. Disease Induced by the Vaccine Organism or Contamination

The vaccine organism in a “live” attenuated vaccine needs to replicate to induce an effective immune response. Vaccine organisms are sufficiently attenuated to not induce specific clinical signs related to the organism in a healthy, immunocompetent cat. In animals with an acquired or congenital immunodeficiency, the vaccine organism might cause clinical signs of an infection against which the animal was supposed to become protected. For example, it has been suggested that immunization of pregnant cats with an attenuated FPV vaccine could lead to cerebellar hypoplasia in the fetus [140], although confirmatory testing was not carried out in the described cases. Generally, modified live FPV vaccines are not licensed for use in pregnant animals. Attenuated strains might also be too virulent for kittens in their first weeks of life. For this reason, contact of colostrum-deprived kittens with recently MLV-vaccinated animals, that might be shedding vaccine virus, is best avoided and live attenuated vaccines should not be administered to kittens younger than four weeks of age. It also has been discussed that in some cases MLV FCV vaccines could have been the cause of FCV outbreaks in cat colonies [141,142,143], but this rarely seems to be the case.

Mucosal intranasal vaccination against respiratory pathogens (e.g., *Bordetella bronchiseptica* vaccine) might evoke mild upper respiratory tract signs. Additionally, if viruses (especially FHV and FCV) in a parenteral vaccine inadvertently come into contact with mucous membranes, clinical signs of upper respiratory tract disease might develop. This might occur if vaccination liquid leaks onto the skin and is licked by the cat or if the vaccine is inappropriately aerosolized during drawing up of the dose from the vial or during injection. In one study, RT-PCR was used to amplify a 235 bp hypervariable region of the FCV genome obtained from vaccinated cats with clinical signs of FCV infection and the sequences were compared to the sequences from three attenuated vaccine viruses. The sequences derived from the vaccine failure cats fell into two categories. Most were distinct (21.33–38.00% distant) from vaccine virus sequences and thus were likely field viruses. However, in some cases, sequences were sufficiently similar to vaccine sequences (0.00–5.33% distant) to suggest that the isolate might have originated from the vaccine strain. However, it was unproven that these cases occurred due to improper administration of the vaccine [144]. Transient shifting lameness due to polyarthritis, which is sometimes seen following FCV infection, in some cases might be due to replication of the vaccine virus with an immune response in the affected joints [39,40].

In rare cases, contamination of the vaccines can lead to severe vaccination-associated diseases. MLV vaccines are considered more susceptible to contamination than inactivated vaccines, especially when vaccines are produced in large numbers and in different production lines. Infected calf serum [145,146] or infected cell cultures [147,148,149,150] can also pose a risk for contamination. However, generally, compliance with good manufacturing practice guidelines can significantly reduce the risk of contamination [151,152,153].

## 8. Lack of Efficacy

Under some circumstances, lack of efficacy is also considered a VAAE. The majority of these are likely to reflect inappropriate storage and administration of the vaccine which is considered poor ‘vaccine husbandry’ [5,154], or use of vaccines in animals unable to respond to them, such as those with high levels of MDA or with immunosuppression. However, the efficacy of batches of vaccines is strictly controlled by the manufacturer and licensing authorities.

## 9. Conclusions

In cats, VAAEs appear to rarely occur. However, limited information on the type and prevalence of VAAEs in cats is available, with the exception of the induction of FISS. Lethargy, with or without fever, is the most commonly diagnosed VAAE. Risk for VAAEs is greater in cats up to one year of age. One factor determined to be associated with an increase in risk was the number of concurrently administered vaccines or the total vaccine volume administered. Therefore, it is recommended to limit the number of vaccine antigens inoculated concurrently to a cat per veterinary visit to further minimize the already low risk of an adverse event.

To specifically prevent FISS development, several recommendations should be followed.

- Vaccination of cats provides essential protection and should not be stopped because of the risk of FISS. It is important to realize that vaccines are not the only injectable medical products associated with FISSs.
- An individually tailored vaccination schedule is important for each cat. Cats should be vaccinated only as often as necessary in accordance with current guidelines.
- Mucosal intranasal vaccines are preferred over injectable vaccines, if available.
- Among injectable vaccines, there is insufficient information to make definitive recommendations on the preferred vaccine type.
- Vaccines should be brought to room temperature prior to administration but should not be kept unrefrigerated for hours.
- Multi-dose vaccine vials should not be used in cats.
- Subcutaneous injection is preferred to intramuscular injection.
- Vaccines with a long duration of immunity are preferred over those with a short duration of immunity.
- Appropriate sites for injections should be selected. The interscapular region as well as the lateral thoracic wall should generally be avoided for any injection. Vaccines should be injected at sites where any subsequent mass could be easily surgically removed, preferably distally in a limb.
- Generally, any skin or subcutaneous mass in a cat requires further diagnostics. Specifically (the “**3-2-1**”-rule), thorough post-vaccination monitoring should be performed. Any lump at the site of injection that is still present **three** months after vaccination, or that is larger than **two** cm in diameter, or that is increasing in size **one** month after vaccination, should be surgically removed and investigated through histopathology.

ABCD recommends that veterinarians carry out a risk-benefit analysis for each vaccine in order to avoid unnecessary vaccination of cats, to monitor the injection site, and report all VAAEs to the manufacturer and/or to their competent authority. The content of this paper is a combination of two ABCD guidelines that will continue to be updated regularly on the ABCD homepage (www.abcdcatsvets.org, accessed on 2 August 2023) as new data become available.
